# Flower development and a functional analysis of related genes in *Impatiens uliginosa*

**DOI:** 10.3389/fpls.2024.1370949

**Published:** 2024-03-25

**Authors:** Haihao He, Xinyi Chen, Tianye Wang, Xiaoli Zhang, Zedong Liu, Suping Qu, Zhijia Gu, Meijuan Huang, Haiquan Huang

**Affiliations:** ^1^ College of Landscape Architecture and Horticulture Sciences, Southwest Research Center for Engineering Technology of Landscape Architecture (State Forestry and Grassland Administration), Yunnan Engineering Research Center for Functional Flower Resources and Industrialization, Research and Development Center of Landscape Plants and Horticulture Flowers, Southwest Forestry University, Kunming, Yunnan, China; ^2^ Flower Research Institute, Yunnan Academy of Agricultural Sciences China, Kunming, China; ^3^ Key Laboratory for Plant Biodiversity and Biogeography of East Asia, Kunming Institute of Botany, Chinese Academy of Sciences, Kunming, China

**Keywords:** *Impatiens uliginosa*, floral development, SEM, floral development genes, floral development model, function of IuAP1 and IuDEF

## Abstract

*Impatiens uliginosa* is a plant of the *Impatiens*, with peculiar flowers. In this study, we combined morphogenesis and molecular biology to explore its development of flowers. An analysis of basic observational data and paraffin sectioning showed that it took approximately 13 d for the floral organs to differentiate. An analysis of the development of inflorescences and floral organs by scanning electron microscopy showed that the inflorescence of *I. uliginosa* is a spiral raceme. The floral organs differentiated in the following order: lateral sepals (Ls), posterior sepal (Ps), anterior sepals (As), anterior petal (Ap), lateral petals (Lp), stamens (St) and gynoecium (Gy). *I. uliginosa* was found to have four sepals, and the connate stamens are caused by the fusion and growth of filament appendages. The results of fluorescence quantification and virus-induced gene silencing showed that *I. uliginosa* had its own unique model for flower development, and there was functional diversity of *IuAP1* and *IuDEF*. Among them, *IuAP1* controls the formation of bract s (Br), regulates the morphogenesis of posterior sepal, controls the anthocyanin precipitation of the anterior petals and the formation of lateral petals. *IuDEF* regulates the morphogenesis of lateral sepals, the length of development of the spur, and controls the size of yellow flower color plaques of the lateral petals. In this study, the process of flower development and the function of flower development genes of *I. uliginosa* were preliminarily verified. This study provides basic guidance and new concepts that can be used to study the development of Impatiens flowers.

## Introduction

Flowers bear the important mission of sexual reproduction. There are approximately 250,000 species of wild flowering plants (Krizek and Fletcher, 2005). Flowering is one of the most important behaviors for plant reproduction and continuation of offspring, and it is also an important condition for the formation of plant diversity. to mature plant through the continuous differentiation of organs. When the plant develops to a certain stage, the combination of endogenous and environmental signals induces flower development. There are many factors that determine flower development, including plant nutrition level, plant hormone level, flowering inducing factors (photoperiod, temperature cycle and vernalization, etc.). The characteristics of floral meristem and floral organ primordia determine the number, speed and type of floral organs during flower development, and floral development genes regulate the type and morphogenesis of plant floral organs (Parcy et al., 1998; Immink et al., 2012).

All complete flowers have a calyx, petals, stamens and pistils, and they develop from outside to inside. Thus, the flowers are divided into 1-4 whorls of floral organ structure. The flower organs of *Impatiens* are peculiar in their morphological differentiation. The types of flower organs include sepals; both posterior and anterior petals; and lateral petals, stamens and pistils. The sepals are divided into lateral sepals and anterior sepals. A study on the development of *Impatiens* and *Hydrocera triflora* flowers showed that the order of flower organ development was the lateral, posterior, and anterior sepals; anterior and lateral petals; and the stamens and gynoecium (Janssens et al., 2012). This study led to the deduction that the lateral sepals, posterior sepals, and anterior petals of *Impatiens* combine to develop the first round of floral organs, the second whorl of anterior petal and lateral petals, the third whorl of stamens, and the fourth whorl of gynoecium.

With the discovery of ABC genes and the analysis of their function, the ABC model of flower development was constructed. The discovery of class D and E genes led to the formation of the most well-known ABCDE model (Ng and Yanofsky, 2000; Theissen, 2001; Bowman et al., 2012; Chen et al., 2018). However, there were few and no systematic studies on flower development genes and flower development models in Impatiens, respectively. Except for *AP2* (*APETALA2*) among the class A genes (Irish, 2010), the ABCDE genes are primarily derived from the MADS-II (MIKC^C^) gene family. The MIKC^C^ genes are divided into 12 subfamilies (Alexandre and Hennig, 2008; Li et al., 2016; Liu et al., 2018), and they all play different roles in the regulation of flower development.

Class A genes primarily regulate the development of sepals, and the primary members are *AP1* (*APETALA1*) and *AP2*. A study on the moth orchid (*Phalaenopsis amabilis*) showed that the *AGL6* gene can function as a class A gene, which forms a protein complex with class B genes and regulates the development of perianth and the precipitation and accumulation of anthocyanins, controls the color of sepals and petals, and regulates the formation of flower spots (Hsu et al., 2021). *AP2* and its homologous genes play an important role in the regulation of the differentiation and formation of spike meristems in wheat (*Triticum aestivum*), paddy rice (*Oryza sativa*), maize (*Zea mays*) and other plants (Chuck et al., 1998; Lee et al., 2007; Chuck et al., 2008; Lee and An, 2012). *AP3* (*APETALA3*) and *PI* (*PISTILLATA*) are class B genes that regulate the morphogenesis of petals and stamens (Hsu et al., 2015). The PI gene was found to play a role in the differentiation of female and male flowers (Sobral and Costa, 2017). The *AG* (*AGAMOUS*) gene is a class C gene, which primarily regulates stamens and pistils in floral organs, and participates in the control of petal, stamen and pistil boundaries (Theissen, 2001). The overexpression mutants of the *AG* gene showed that the increased expression of this gene could lead to the transformation of petals into stamens (Su et al., 2018), and the deletion of *AG* gene led to the transformation of stamens into petals. The class D gene *AGL11* (*AGAMOUSLIKE11/STK*) is a homologous gene of *AG*. It has been found to regulate the development of ovules and carpels and has been identified as a class D gene (Pinyopich et al., 2003). The *AGL11* gene not only regulates the development of pistils and ovules but also regulates the differentiation of plant calli (Fei et al., 2021).

The class E gene *SEP* (*SEPALLALA*) is a type of auxiliary gene, which forms a tetrameric protein complex with the A, B, C and D genes and promotes the development of floral organs in each round (Hu et al., 2021). The differentiation and development of floral organs requires the participation of *SEP* gene, which plays an important role in the recognition of plant organs (Wang et al., 2015). Studies have shown that the ABCD genes interact with class E genes to form a protein dimer, and then two protein dimers combine to form a protein tetramer structure, which was the theoretical basis for the construction of tetramer phantom for flower development (Baum and Hileman, 2007; Ruokolainen et al., 2010; Seok et al., 2010; Zhou et al., 2016). The tetramer model of flower development shows the regulatory mechanisms of floral organ formation (Theißen and Gramzow, 2016), including 2*AP1 +* 2*SEP*=Se (Sepal), *AP1*+*AP3*+*PI*+*SEP*=Pe (Petal), *AG*+*AP3*+*PI*+*SEP*=St (Stamen), 2*AG*+2*SEP*=Ca (Carpel) and *AG*+*STK*+*SHP*+*SEP*=Ov (Ovule).

*I. uliginosa* is a typical *Impatiens* plant, which was considered to have only two lateral sepals (Yu et al., 2016). The other two anterior sepals degenerated. The characteristics of inflorescence differentiation, the sequence of floral organ development, and the characteristics of floral organ primordia were studied, and the expression of ABCDE genes in floral organs was analyzed. This study explained the types of floral organs, constructed a model for flower development, and hypothesized the tetramer model of flower development. These findings should help future research on the development of *Impatiens* flowers and provide new ideas and methods for the structural development and molecular breeding of *Impatiens*.

## Materials and methods

### Materials

*I. uliginosa* is a typical Impatiens plant, which is an annual or perennial herb, and its inflorescence is a raceme (**Supplementary Figure 1**). Well-developed seedlings were selected for culture. They were cultured with suitable water and fertilizer management, and no physical or chemical experiments were performed on the seedlings. The plants were left to develop normally. The materials were planted in the tree garden of Southwest Forestry University (Kunming, China).

### Methods

#### Flower development stage division and duration statistics

**Figure 1:**
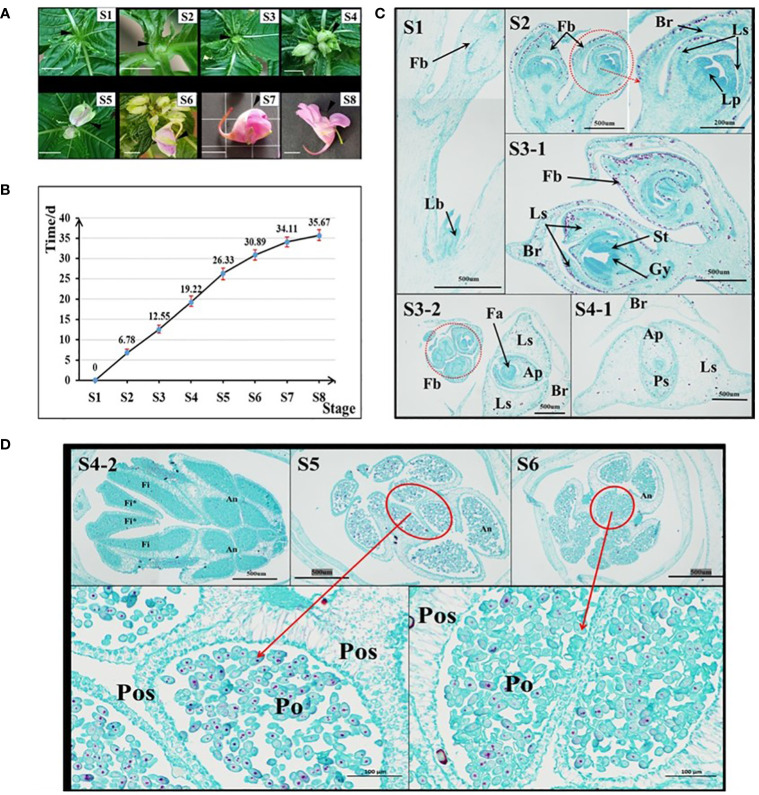
Analysis of the characteristics of flower development time in *I. uliginosa.* **(A)** Reference diagram of period S1-S8 of *I. uliginosa*. The ruler is 1 cm. **(B)** Statistical chart of the duration from S1 to S8. **(C)** Paraffin sections of the S1-S4 period. S3-2 and S4-1 are horizontal slices, while the rest are longitudinal slices; Fb, Flower buds; Lb, Leaf bud; Br, Bract; Ls, Lateral sepals; Ps, Posterior sepal; Ap, Anterior petal; Lp, lateral petals; St, Stamens; Gy, Gynoecium; Fa, floral apex. **(D)** Paraffin sections of flower buds in the S4-S6 period. Fi, Filament; Fi*, Filament appendage; An, Anther; Po, Pollens; Pos: Pollen sac wall.

In the *I. uliginosa* flowering stage, the organs were divided as shown in **Figure 1A**. S8 stage: when the first flower was in full bloom. S7 stage: when the first flower bud was ready to bloom; S6 stage: when the pedicel of the first bud was approximately 1 cm; S5 stage: when the first bud was completely detached from the bract’s protection; S4 stage: when the first bract was semi-protected; S3 stage: when the inflorescence was exposed; S2 stage: when the inflorescence was removed from the top leaf; S1 stage: when the leaf bud was about begin its differentiation into flower buds. Images were taken of the development of inflorescences of plants during these eight periods to serve as a reference. Nine plants with the developmental status of S1 stage were found, marked, and recorded on the 0 d. The time when each plant developed to each stage was recorded.

#### Paraffin sectioning

The inflorescences in the S1-S3 period and the first bud in the S4-S6 period were sampled based on the division of the period in Section 1 (Flower development stage division and duration statistics). The sample was fixed with formaldehyde, acetic acid and ethanol (FAA) for 48 h and then dehydrated. The routine paraffin section method was used for slicing and observation. The samples were dehydrated using a dehydrator (Donatello, Diapath, Martinengo, Italy) and Entrapment (Encapsulation machine, JB-P5). The tissues were sectioned on a Leica RM2016 slicer (Wetzlar, Germany). Tissue sample exhibition film (Organizing a stall, KD-P). After the steps listed above had been completed, the tissues were incubated at 60 °C until the film had dried. After the tissues had been stained with Safranin 0-Fast Green, the images were observed by light microscopy (Nikon, Tokyo, Japan) and analyzed using an imaging system (Nikon).

#### Scanning electron microscopy

FAA stationary liquid was used to sample each period before the S5 period (complete inflorescence). After dehydration, an anatomical needle was used for preliminary dissection to remove unnecessary organs and impurities. The critical drying point was determined using a critical point dryer (EMS 850; Electron Microscopy Services, Hatfield, PA, USA). The drying was completed, and secondary dissection was performed under an anatomical microscope. Gold was then sprayed by a 108 Auto Sputter Coater (Cressington Scientific Instruments, Watford, UK), and the tissues were finally photographed and analyzed by a Zeiss Sigma 300 SEM (Zeiss, Jena, Germany).

#### Gene screening and identification

The ABCDE gene was screened, and the CDS sequence of the gene was obtained using the *I. uliginosa* flower transcriptome data obtained in previous research by this group (Majorbio Bio-Pharm Technology Co., Ltd. Shanghai, China). NCBI (https://www.ncbi.nlm.nih.gov/) was then used for a bidirectional BLAST alignment at NCBI (https://www.ncbi.nlm.nih.gov/). This alignment was used to obtain potentially similar sequences and identify genes. The class A genes *IuAP1* and *IuAP2*, class B genes *IuDEF* and *IuGLO*, an *IuAG* gene (class C), a class D gene (*IuAGL11*) and two class E genes (*IuSEP1* and *IuSEP3*) were obtained (**Supplementary Annex 1**).

#### Fluorescence quantitative analysis

The floral organs of the S5 stage were sampled and divided into seven types of floral tissue, including lateral sepals, posterior sepals, anterior sepals, anterior petals, lateral petals, stamens, and the gynoecium among others. One benefit of conducting research in the S5 stage is that the floral organs are easily peeled off. The floral organs had not fully matured, and the functional genes involved in flower development were still partially active. The total RNA of each part was extracted, detected (**Supplementary Figure 2**), and then reverse transcribed into cDNA. The fluorescent quantitative primers were designed by NCBI (https://www.ncbi.nlm.nih.gov/) and sent to the company for synthesis (**Supplementary Figure 4**). The sequence of primers is shown in **Supplementary Information 2**. Finally, the commonly used value (CP) of each experimental group was obtained by a fluorescence quantitative experiment, and the relative expression of each gene in each flower organ was calculated using the following formula: EQ (relative expression) = 2 ^ (- Δ CP). An expression heat map was constructed using the quantity of relative expression, and the significance of the average difference (positive value) was used to express the importance of the gene. In addition, the genes were marked by color in the floral organ plane model of *I. uliginosa*. Darker colors indicate higher levels of expression.

#### Gene cloning

cDNA was obtained by the reverse transcription of RNA as the template, and primers were designed and synthesized. PCR was used to amplify the full length of *IuAP1* (F: 5-ATGGGGAGAGGGAGAGTG-3) and *IuDEF* (F: 5-ATGGCCAGAGGAAAGATCCAG-3). The reaction system was as follows: cDNA template 1 ul, primers 1 ul, Taq PCR StarMIX with Loading Dye 2 × Taq PCR StarMIX with Loading Dye, 10 ul; and sterile ddH2O, 7 ul. The reaction procedure was as follows: pre-denaturation for 5 min at 94°C, denaturation at 94°C, annealing at 56 °C, annealing at 56°C, extension at 72°C for 48 s, 35 cycles, extension at 72°C for 5 min and preservation at 4°C. The PCR products were obtained, and a Biomed gel recovery kit was used to recover the DNA. It was then ligated to the pMDTM 19-T vector, transformed into Escherichia coli *DH5α*, and verified by sequencing.

#### Virus silencing gene technology

According to the pTRV2 vector map, BamH I-Xho I was designed as the insertion site, and the primers were synthesized. The target fragments of *IuAP1* (F: 5-GAAGGCCTCCATGGGGATCCGAACAAGATCAATAGGC-3; R:5-GGACATGCCCGGGCCTCGAGGTCTTCTCTATCTCCTTG-3) and *IuDEF* (F: 5-GAAGGCCTCCATGGGGATCCACCGGCAAACTCCATG-3; R:5-GGACATGCCCGGGCCTCGAGGTGAATCTCAATCTG-3) were amplified. The target gene was ligated to the vector by double enzyme digestion and homologous recombination and then transformed into DH5 α for propagation. The positive clones were sequenced, and the plasmids were extracted. The positive clone plasmid was transformed into Agrobacterium tumefaciens GV3101, and positive detection and expanded culture were conducted. The cells were grown to an OD600 of 0.6~0.8, and the plants were infected by soaking and injection.

## Results

### Analysis on the time of floral organ differentiation of *I. uliginosa*

*I. uliginosa* experienced approximately 36 days from S1 to S8 (**Figure 1B**). S1-S2 took approximately 7d; S2-S3 took approximately 6d; S3-S4 took approximately 6d; S4-S5 took approximately 7d; S5-S6 took approximately 5 d; S6-S7 took approximately 3 d; and S7-S8 took approximately 2 d. The entire process took 36 days in total.

The results of paraffin sections observations at the S1-S6 stages showed the development of floral organs in *I. uliginosa* (**Figures 1C, D**). At the S1 stage, the terminal bud primordia of *I. uliginosa* were transformed into the inflorescence bud primordia, and the lateral bud primordia still differentiated as normal for the leaf bud primordia. During the S2 period, the inflorescence further differentiated. The petal primordium was differentiated from the first bud, and the perianth primordium was completely differentiated at this time. Combined with the time of flower development and fruiting, the perianth primordium differentiated within 7 d. The pistil primordium differentiated at the S3 stage, which indicated that the time it took the time of stamen and pistil to differentiate was approximately 6 d, and the flower organ primordia took approximately 13 d to differentiate.

At the S4 stage, the size of the Flower bud was larger than that of the bract (**Figure 1B** [S4-1]); the protection of the bract was weakened, and the filament appendages almost fused and covered the carpels. The pollen sac of the anther was closely organized, which was the initial stage of pollen division. At the S5 stage, part of the Pollen proliferation completed its morphological formation, and some pollen grains were proliferating and differentiating compared with the S6 period. The pollen at S6 stage filled the pollen sac, and there were few pollen grains for proliferation and differentiation. The section pictures of S5 and S6 showed that the thickness of the outer wall of the pollen sac was much thicker than that of the inner wall, which could more effectively protect the development of pollen. The pollen took more than 11 d to develop, which comprised approximately one-third of the whole time of flower development. This showed that A spends most of its time on reproductive differentiation.

#### Characteristics of the differentiation of inflorescence tissue of *I. uliginosa*

**Figure 2:**
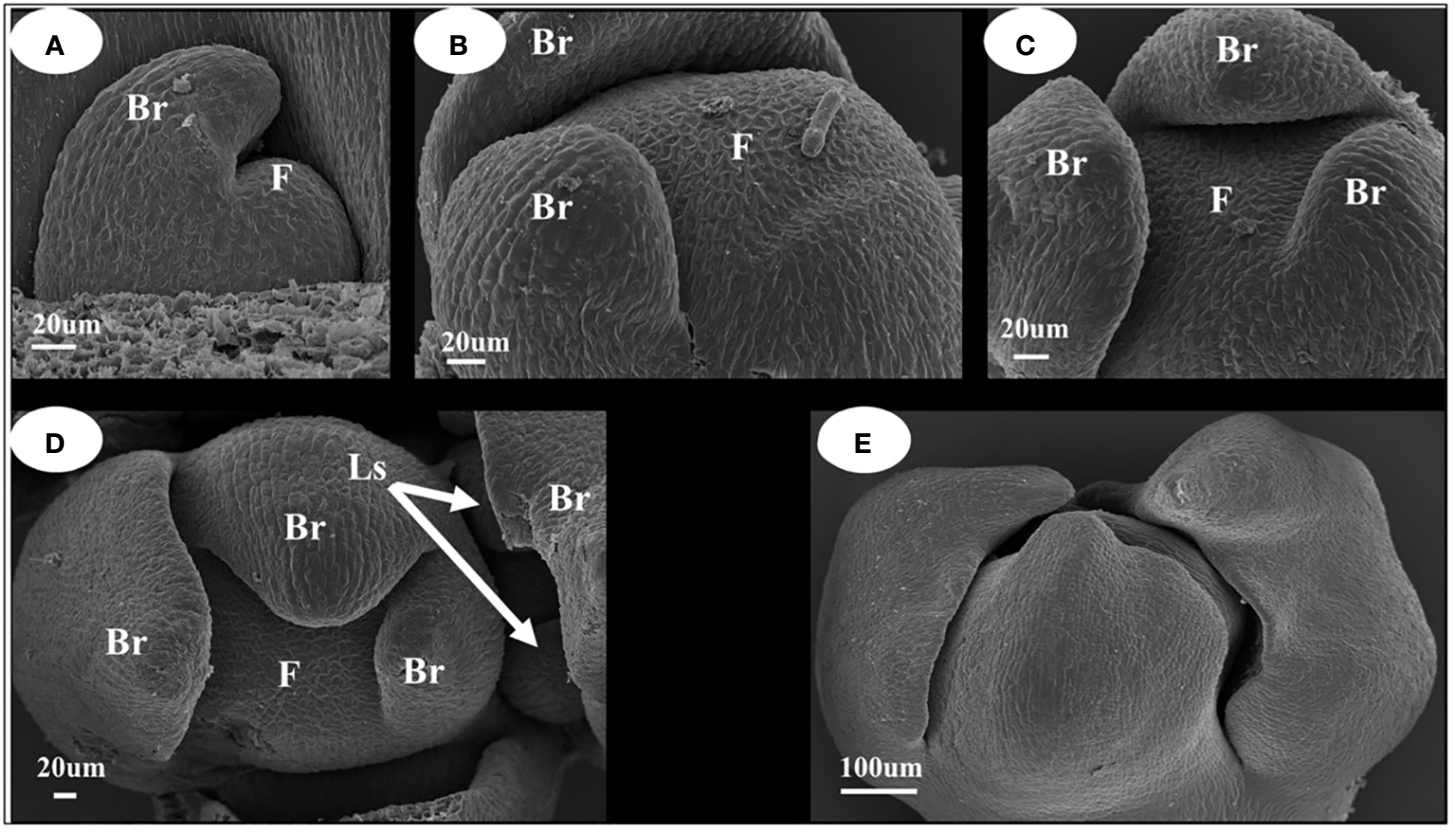
Inflorescence development map of *I. uliginosa*. Br, Bract; Ls, Lateral sepals; F, Inflorescence apex. **(A–C)** Characteristics of Bract primordium differentiation. **(D)** Initial differentiation diagram of Sepals primordium. **(E)** Early inflorescence diagram.

When the inflorescence differentiated (**Figure 2**), the stem tip leaf bud was transformed into an inflorescence owing to the differential transformation into inflorescence, and the leaf primordium transformed into a bract primordium (**Figures 2A–C**). When the bract primordium differentiates into more than three pieces, the axillary part of the bract differentiates into lateral sepal primordia (**Figure 2D**). From the characteristics of the position of differentiation of the bract primordium, it can be concluded that the *I. uliginosa* inflorescence differentiates into a spiral structure. Thus, its inflorescence was a spiral raceme. The tip of the whole inflorescence was surrounded by pre-developed bracts (**Figure 2E**), which can more effectively protect the apical meristem of the inflorescence. The flower organ primordia began to differentiate when the bracts completely covered the flower apical meristem. The differentiation of inflorescences and floral organ primordia were closely combined and conducted synchronously.

#### Characteristics of the differentiation of floral organ primordia in *I. uliginosa*

**Figure 3:**
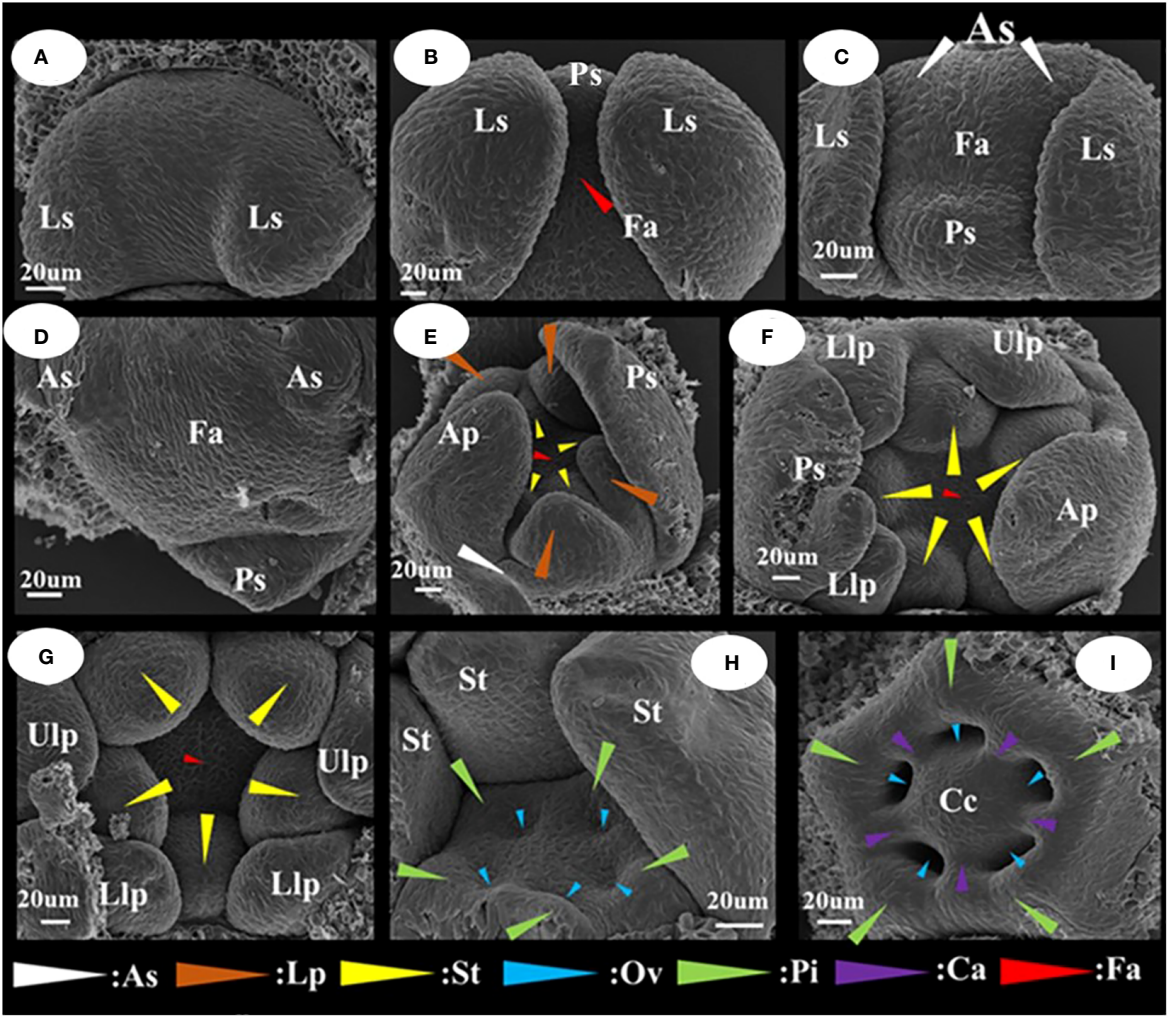
Floral organ primordium development map of *I. uliginosa*. **(A)** Primordium of Lateral sepals. **(B)** Primordium of Posterior sepal. **(C, D)** Primordium of Anterior sepals. **(E)** Primordium of Anterior petal and Lateral petals. **(F, G)** Primordium of Stamens. **(H–J)** Primordium of Gynoecium. Ls, Lateral sepals; Ps, Posterior sepal; As, Anterior sepals; Ap, Anterior petal; Lp, Lateral petals; Ulp, Upper lateral petals; Llp, Lower lateral petals; St, Stamens; Gy, Gynoecium; Fa, floral apex; Pi, Pistils; Ov, Ovary; Ca, Carpels; Cc, Central column.

The differentiation of floral organ primordia and the developmental stage characteristics of *I. uliginosa* were readily apparent (**Figure 3**). They can be divided into seven stages, which were differentiation of the lateral sepal primordium; differentiation of the posterior sepal primordium; differentiation of the anterior sepal primordium; differentiation of the anterior petal primordium; differentiation of the lateral petal primordia; differentiation of the stamen primordia; and differentiation of the gynoecium/carpel primordia. The side of bract was taken as the upper side. When the lateral sepals differentiated, the width of the upper side was significantly larger than that of the lower side (**Figure 3A**). The anterior sepal primordia differentiated behind the posterior sepals, and two anterolateral primordia developed simultaneously and were the same size (**Figures 3B–D**). When the anterior petal primordia differentiated (**Figure 3E**), the rate of growth of the anterior petal was higher than that of the anterolateral sepals. The upper lateral petals and lower lateral petals differentiated simultaneously, and with the growth, the upper lateral petals grew less than the lower lateral petals. Finally, the upper lateral petals were much smaller than the lower lateral petals. The primordia of stamens and pistils occur, in turn, with a cardinal number of five (**Figures 3F–I**). The pistil primordium occurs around the terminal tissue of the flower and forms a regular pentagonal shape. The pistils were congenitally connate, and the carpel walls were formed in the connate part, which divided the seed pod into five small chambers to form an ovary. The number and location of the differentiation of flower organ primordia ensured that the flower developmental process was in a state of left and right symmetry.

*I. uliginosa* differentiated a total of 20 floral organs, which were divided between the upper and lower lateral petals (regardless of tangent division, tangent division into two lateral sepals, two stamens, and two pistils for a total of six floral organs). There were nine upper floral organs and five lower floral organs. There were approximately twice as many upper floral organs as that of the next time. The larger perianth segment in the lower part was the reason to maintain the upper and lower structures of the flower and ensure that the floral organs were symmetrical.

#### There are four sepals in *I. uliginosa*

**Figure 4:**
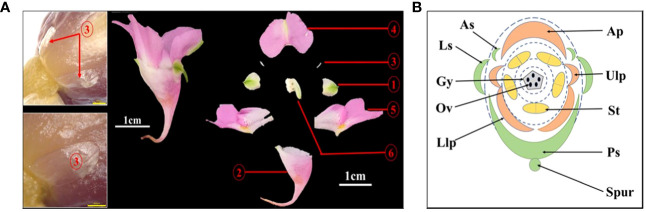
Diagram of the anatomy and plane models of *I. uliginosa*. **(A)** Flower anatomy diagram.1: Lateral sepals; 2: Posterior sepal; 3: Anterior sepals; 4: Anterior petal; 5: Upper and Lower lateral petals; 6: Androgynous group. **(B)** Flower plane model diagram. Ls, Lateral sepals; Ps, Posterior sepal; As, Anterior sepals; Ap, Anterior petal; Ulp, Upper lateral petals; Llp, Lower lateral petals; St, Stamens; Gy, Gynoecium.

In the Flora of China and Balsaminaceae of China, two sepals of *I. uliginosa* were recorded. However, during the process of development of the floral organ primordia of *I. uliginosa*, there were two anterior sepal and four sepal primordia. An ultra-depth-of-field three-dimensional microscope (**Figure 4A**) was used to observe the developmental position of the anterior sepals of the flower bud at the S6 stage, and two anterior sepals were produced and developed. The anterolateral sepals of *I. uliginosa* were small and enclosed by lateral sepals, so they were not easily observed. Based on the anatomical map of the flower, a plane diagram of the flower of *I. uliginosa* was established (**Figure 4B**). The structural characteristics of its four-whorled floral organs and the characteristics of the cardinal number of floral organs of five were established.

#### Characteristics of the filament appendages and stamens of *I. uliginosa*

**Figure 5:**
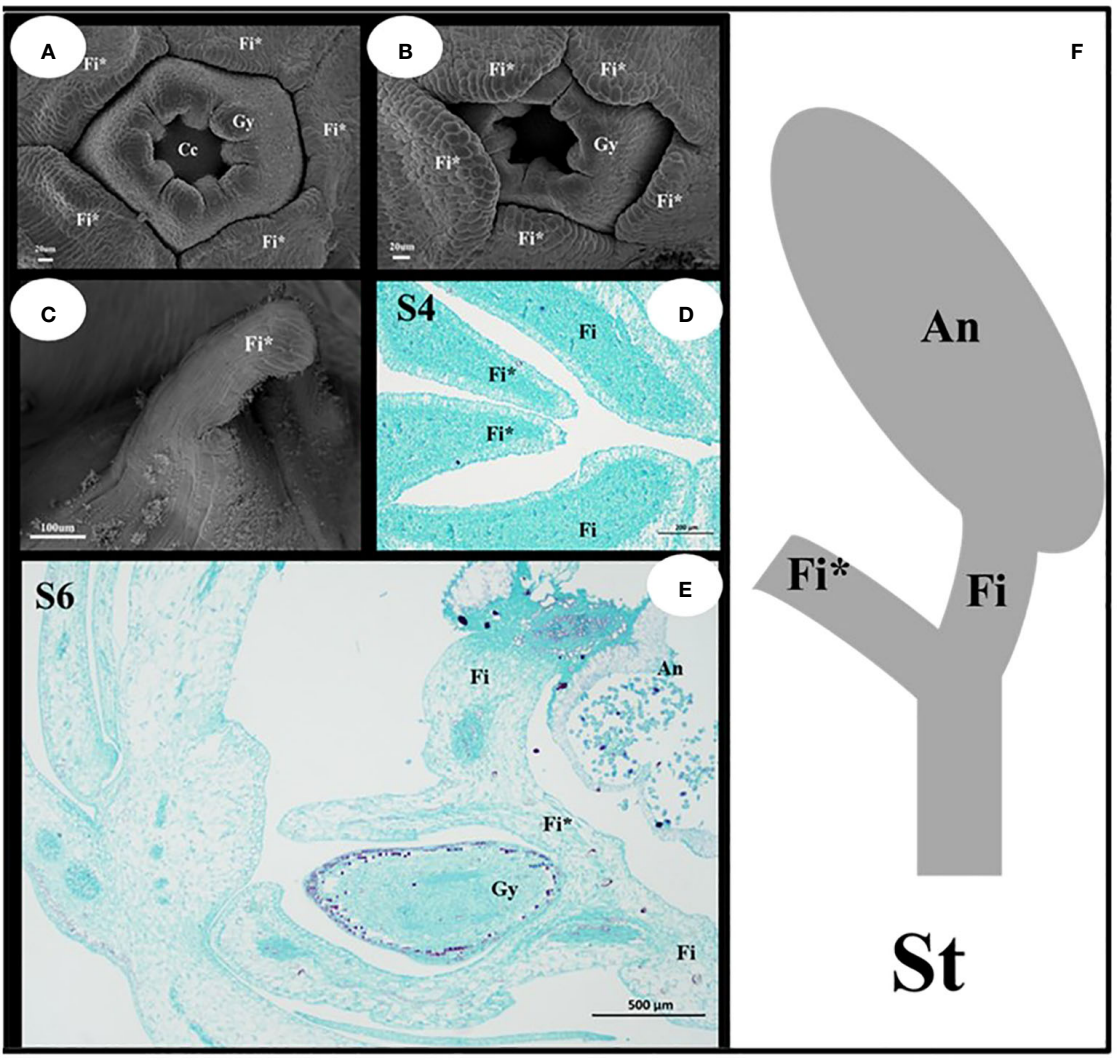
Structural characteristics of the filament appendages and stamens. **(A, B)** Characteristics of Filament appendages. **(C)** Filament appendages connate. **(D, E)** Location characteristics of Filaments and Filament appendages. **(F)** Mature Stamen model diagram. Fi, filaments; Fi*, filament appendages; Gy, gynoecium; An, anthers; St, stamens.

The filament appendages are filament structures that have differentiated from filaments (**Figure 5**). These appendages differentiate from the filaments after the pistils appear, and their growth rate was higher than that of the pistils (**Figures 5A, B**). The early filament appendages were in a free state, and with further growth and development, they merged with each other to form a conical hat structure that covers the pistil (**Figure 5C**). The results of paraffin sectioning at the S4 stage showed that the filament appendages almost completely covered the pistil during this period (**Figure 5D**). As the pistil grew during the S6 stage, the filament appendages were squeezed into a lid-like shape, which covered the pistil or carpels (**Figure 5E**). The filament appendages fused and grew, which caused a change in the stamens from a congenital free state to a symbiotic state. The filament structure of each stamen was bifurcated (**Figure 5F**). One end was connected to the anther, and the other end was connate with the other four filament appendages that formed a protective wall around the pistil.

#### Analytical expression of the quantitative expression of fluorescence

**Figure 6:**
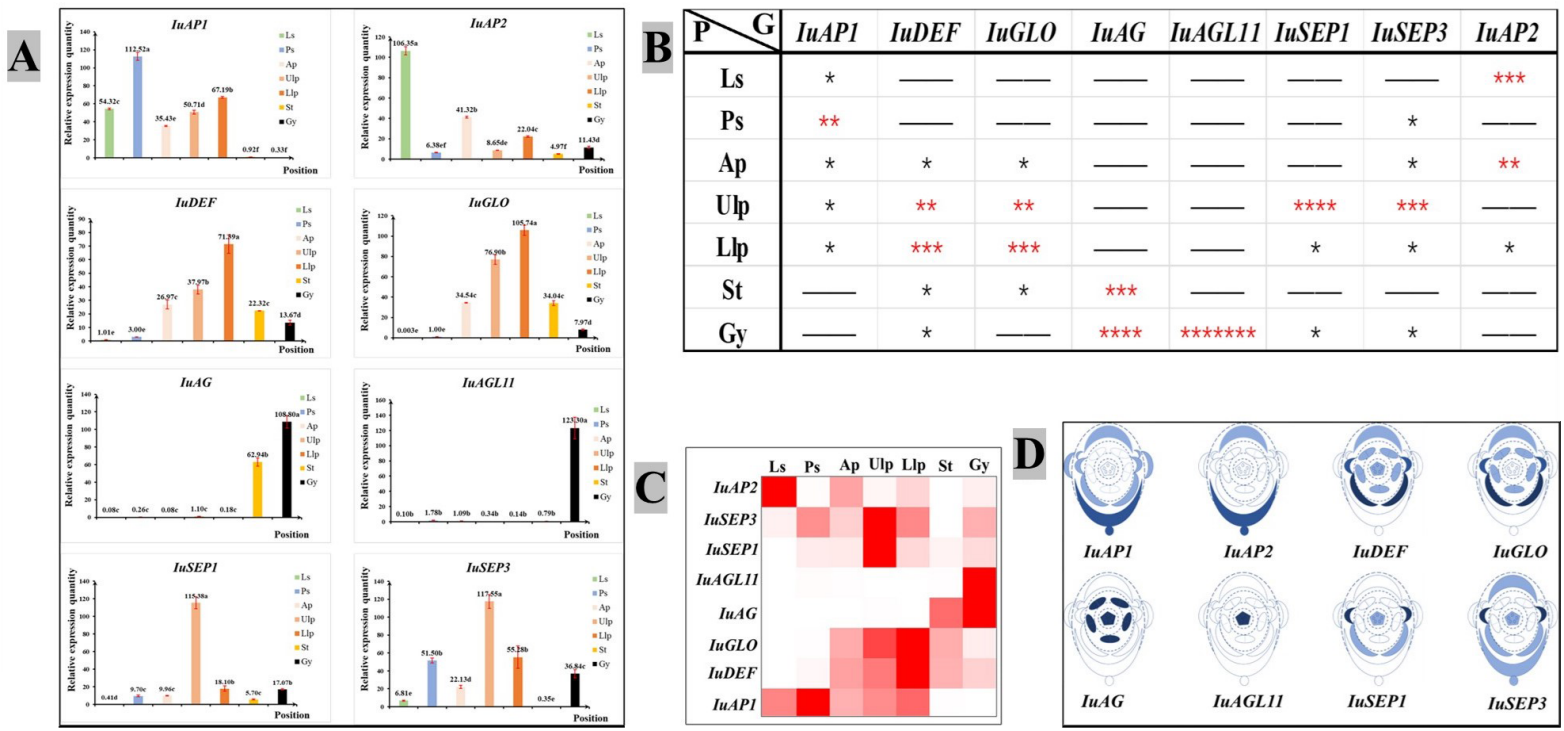
Chart of the analytical expression of the quantitative fluorescence. **(A)** Analysis of relative gene expression. **(B)** Analysis of gene importance. * significant. More asterisks indicate enhanced usefulness of the gene. **(C)** Expression of the heat map. **(D)** Analysis of the location of gene expression. Ls, Lateral sepals; Ps, Posterior sepal; As, Anterior sepals; Ap, Anterior petal; Ulp, Upper lateral petals; Llp, Lower lateral petals; St, Stamens; Gy, Gynoecium.

An analysis of the quantitative expression of fluorescence revealed differences in the expression of the ABCDE genes (**Figure 6A**). The class A gene *IuAP1* was expressed in the tepals, and it was expressed the most highly in the posterior sepal. However, it was barely expressed in the pistils and stamens. *IuAP2* was expressed the most highly in the lateral sepal, followed by the anterior petal. Of the Class B genes, *IuDEF* was primarily expressed in the anterior and lateral petals but expressed at lower levels in the stamen and pistil. *IuGLO* was barely expressed in the lateral and posterior sepals. However, it was expressed the most highly in the lateral petal. The *IuAG* gene was only expressed in the stamen and carpel, while *IuAGL11* was only expressed in the carpel. The two *IuSEP* genes were expressed in almost all the floral organs, and their levels of expression were the highest in upper lateral petals. In summary, the selected genes from ABCDE were suitably expressed, and it was confirmed in more detail that they are functional genes that regulate the development of flowering in *I. uliginosa*.

#### Analysis of the importance of ABCDE genes

A visual heat map of gene expression was constructed based on the relative level of expression of genes (**Figure 6C**), and the significance of each gene in each floral organ was analyzed using the significant difference of average value (**Figure 6B**). Based on the analysis described above, they were marked in the plane model of *I. uliginosa* (**Figure 6D**). The results showed that the class A genes (*IuAP1* and *IuAP2*) were primarily expressed in the lateral sepals, posterior sepals, anterior petals and lateral petals. The Class B genes (*IuDEF* and *IuGLO*) were primarily expressed in the anterior petals, lateral petals and stamens. The Class C gene (*IuAG*) played a major role in the stamens and carpels, while the Class D (*IuAGL11*) genes acted on the carpels or ovules. The Class E genes (*IuSEP1* and *IuSEP1*) were expressed in almost all the parts but had a stronger regulatory effect on the upper lateral petals.

#### Functional analysis of IuAP1 in *I. uliginosa*

**Figure 7:**
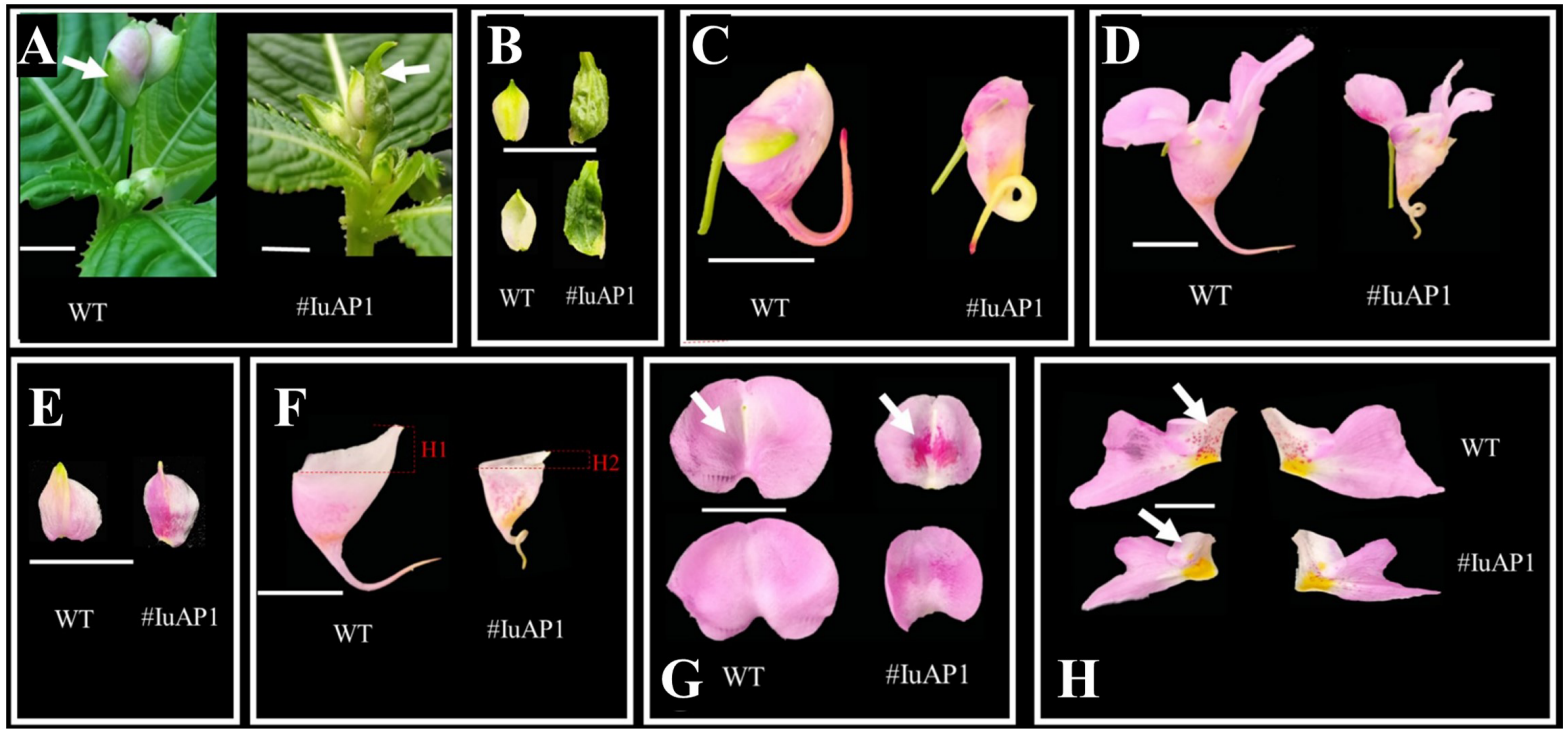
The effect of *IuAP1* on the development of *I uliginosa*. **(A, B)** Characteristics of Bract development. **(C, D)** The difference of flower morphology. **(E)** Differences in the morphology of Lateral sepals. **(F)** Differences in the morphology of Posterior sepal. **(G)** Differences in the morphology of Anterior petal. **(H)** Differences in the morphology of Lateral petals. WT, Wild type; # IuA1, *VIGS-IuAP1*.

VIGS technology was used to silence *IuAP1* (**Figure 7**), and this silencing led to several traits. The bracts turned into leaves, and the flowers become smaller (**Figures 7A, B**). Local areas of the lateral sepals and Anterior petal were darker (**Figures 7E–G**). The flowers on the posterior sepals changed their spacing and became more curved (**Figures 7C, D, F**). In addition, and the upper length of the posterior sepal was shortened. The number of red spots on the lateral petals decreased (**Figure 7H**), and the flowers became lighter. In summary, *IuAP1* is the key gene that determines the formation of bracts and thus, controls the morphological formation of bracts in *I. uliginosa*. This gene controls the morphological development of flower spacing and the direction of development of flower distances during the process of development of the posterior sepals. During the process of formation and accumulation of flower colors, the mechanism of the formation of the anterior petals and lateral petals varied. *IuAP1* promotes the formation of red spots in the anterior petals and inhibits the coloring and accumulation of red spots in the lateral petals.

#### Functional analysis of IuDEF in *I. uliginosa*

**Figure 8:**
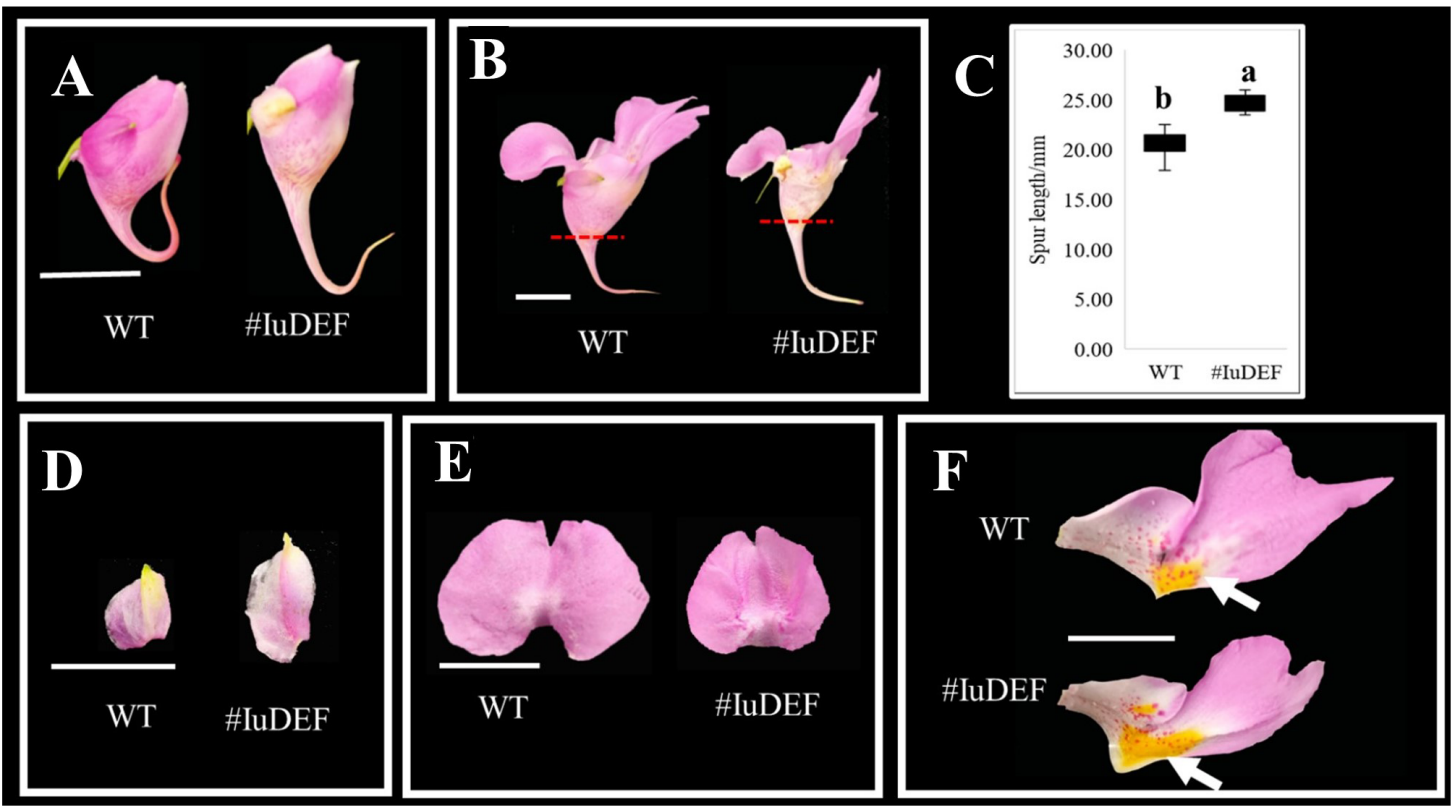
Effect of *IuDEF* on the development of *I. uliginosa*. **(A, B)** The difference of flower morphology. **(C)** Spur length. **(D)** Differences in the morphology of Lateral sepals. **(E)** Differences in the morphology of Anterior petal. **(F)** Differences in the morphology of Lateral petals. WT, Wild type; # IuAP1, *VIGS-IuDEF*.

*IuDEF* is a class B gene in flower development, and its silencing resulted in a significant increase in the spur length of the posterior sepal (**Figures 8A–C**) and an increase in the size of lateral sepals (**Figure 8D**). More petalizing characteristics were visible, and there was no significant difference in morphogenesis between the anterior petal and lateral petals (**Figures 8D, E**). In addition, the silencing of *IuDEF* resulted in areas of larger yellow spots on the lateral petals (**Figure 8F**).

Therefore, *IuDEF* not only regulates the morphogenesis of lateral sepals; it also controls the size of the lateral sepals and inhibits the enlargement of lateral sepals. The silencing of this gene showed that it also regulates the Supr elongation of the posterior sepal. In addition, *IuDEF* regulates the accumulation of yellow petals on the lateral petals, and its silencing leads to an increase in the areas of yellow spots. In contrast, it shows that *DEF* inhibits the accumulation and coloration of anthocyanins in the yellow spots to some extent.

## Discussion

### Evolutionary analysis of *Impatiens*

There are more than 1,000 species of *Impatiens* in the world (Luo et al., 2022). Studies on their evolution and taxonomy show that the number of sepals is one of the signs of evolution. Combined with the palynological and carpel quantitative characteristics, the widely recognized evolutionary relationship was four Sepals, four Carpels, three-Groove germination holes; four Sepals, five Carpels, three-Groove germination holes; and two Sepals, five Carpels, and four-Groove germination holes (Yu et al., 2016). A study of the differentiation of floral organs of *Impatiens omeiana* showed that all the floral organ primordia were present (Janssens et al., 2012), and the characteristics of differentiation of the floral organs of *I. uliginosa* were highly consistent with the results of a study by Janssens et al. (2012). However, in a study of *Impatiens hawkeri* and *Impatiens niamniamensis* (Caris et al., 2006), the anterior sepal primordia were rudimentary, and there were actually two sepals. Therefore, we hypothesized that the evolutionary direction of impatiens had a relationship with the size of sepals. The evolutionary rules of Impatiens were as follows: (1) Four sepals, with larger anterior sepals, trifurcate pollen, and four carpels. (2) Four sepals, with larger anterior sepals, trifurcate pollen, five carpels. (3) Four sepals, with larger anterior sepals, four-furrow pollen, five carpels. (4) Four sepals, anterior sepals smaller, four-furrow pollen, five carpels. (5) Four or two sepals, anterior sepals primordium developed, four-furrow pollen, five carpels. (6) Two sepals, anterior sepals primordium development, four-furrow pollen, five carpels. (7) Two sepals, anterior sepal primordia rudimentary, four-furrow pollen, five carpels.

### Analysis of the characteristics of floral organ development in *Impatiens*

The development of *I. uliginosa* floral organs belongs to the flower development type of four sepal Impatiens. Compared with the relatively primitive development of *Impatiens* (Janssens et al., 2012), after the development of anterior sepals, it entered into the differentiation of anterior petals, and the anterior petals grew rapidly and exceeded the growth rate of the anterior sepals. Moreover, compared with the Impatiens with four sepals, the Impatiens with two sepals may not differentiate, and the anterior petal may grow more quickly (Caris et al., 2006). The rate of development of the anterior petals of Impatiens with smaller anterior sepals was similar to that of two Impatiens. Therefore, the reduction or degeneration of the anterior sepals may promote the rapid development of the anterior petal, thus, more comprehensively protecting the internal tissue of the flower. It is well known that the pterygoid, stamens and carpels of most Impatiens are syncytial organs. The results showed that before the early development of Impatiens, the primordia of almost all the organs were separated (except for the carpels). *Impatiens is* the same as most plants, and the carpels showed symbiotic characteristics (Endress, 2011). However, the pterygoid and stamens were acquired (Janssens et al., 2012). With the growth of upper lateral petals and lower lateral petals, the basal tissues fused and grew, which resulted in the symbiosis of lateral petals. The symbiotic growth of stamens was very interesting and was caused by the accessory symbiosis of filaments that differentiated from the filaments.

The placenta of Impatiens is considered to be the central placenta (Ramadevi and Narayana, 1989). The placental primordium appeared in the middle stage; the columnar growth was formed in the later stage, and the ovule differentiated on the placental column. Janssens et al. (2012) pointed out that the placenta was formed at the intersection of the carpel diaphragm, but our result was more like a placental column growing from the bottom of the carpel than what they described.

The pollen proliferates and develops after the S4 stage. When it reached the S6 stage, the morphological characteristics of the division were close to maturity, and the pollen was less proliferated and differentiated. This result had important guiding significance for the cross breeding of Impatiens. During the process of cross-breeding Impatiens, the stamens should be removed at approximately the S6 stage.

### Analysis of the flower symmetry of *Impatiens*

In comparison with other studies on the floral organ development of *Impatiens* (Caris et al., 2006; Yu et al., 2010; Janssens et al., 2012), each stage of organ differentiation ensures that the flower is in a state of left and right symmetry. The lower organ of the flower was larger, which makes up for the disadvantage of a small number of organs, and relies on the advantage of gravity to maintain the characteristics of the lower posterior sepal of the flower and the upper anterior petal, which is convenient for pollination by insects.

### Analysis of the self-protective mechanism of the differentiation of floral organs in *I. uliginosa*

A study on the development of inflorescences (Janssens et al., 2012) showed that *I. uliginosa* was a spiral raceme, and the spiral structure was more stable. When the bracts differentiate into the or more pieces and can protect the meristem of floral organs, the sepal primordium begins to differentiate. The stamens begin to differentiate when two lateral sepals are enveloped by internal tissue. The stamens are one of the most important organs for plant reproduction. The pistil began to differentiate when the stamen covers the flower apical meristem. At this time, the closed space formed by the perianth segment can completely protect the internal structure of the flower. When the protective function of bracts was weakened (S4 stage), the filament appendages of stamens grow together to form a cap-like structure to protect the development of pistil. In the flower stamens, the outer wall of the pollen sac was thicker than the inner wall, which reduces the threat of pollen entering from the outside. Each stage of the floral organ differentiation of *I. uliginosa* serves to protect the internal tissue or genetic material of the flower from external damage.

### Analysis of the ABCDE model of flower development of *I. uliginosa*

**Figure 9:**
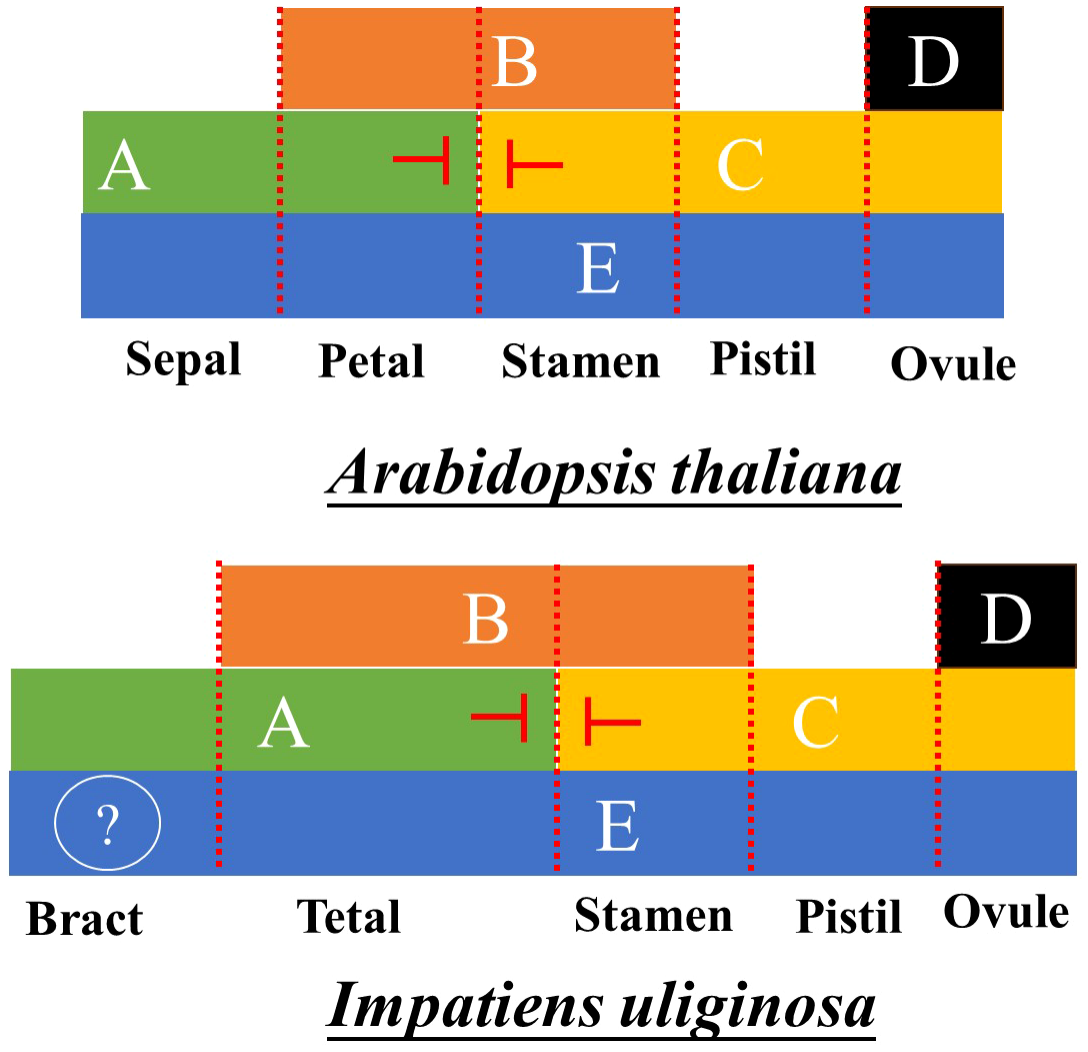
ABCDE model for the Development of *Arabidopsis thaliana* and *I. uliginosa*.

Based on an analysis of the expression of ABCDE genes, the flower development model of *Impatiens* was constructed. Because class AE genes regulate the first round of floral organ development, the ABE genes regulate the petals; the BCE genes regulate the stamens; the class CE genes regulate the pistils, and the class DE genes regulate the ovules (Theißen and Gramzow, 2016). In *Impatiens*, the class A genes primarily played a role in perianth segments, while the class B genes were highly expressed in the anterior petals, lateral petals and stamens. However, combined with the results of *IuAP1* and *IuDEF* silencing, the ABCDE model of flower development of *I. uliginosa* was quite different from that of *Arabidopsis thaliana* (Theißen and Gramzow, 2016). Bracts are directly controlled by class A genes, ABE genes regulate tepals, BCE genes regulate stamens, and DE genes regulate pistils (**Figure 9**). The posterior sepal and sepals cannot really belong to the first round of floral organ structure, which is inconsistent with the hypothesis of Yu (2012). Supported the hypothesis that there were five developmental cardinal numbers of the floral organs of Impatiens (Yu et al., 2016). In this study, we proposed the flower development model of Impatiens and summarized the regulatory genes of the development of each floral organ.

### Functional analysis of the flower development genes of *I. uliginosa*

#### Class A gene

*IuAP1* in *Impatiens* is consistent with that of most plants, indicating that the function of *AP1* in the development of *Impatiens* is conservative and similar to that of other plants (Kaufmann et al., 2010). *AP1* was found to activate the anthocyanin biosynthetic pathway (Li et al., 2020). The posterior sepal and sepals of *Impatiens* belonged to the same whorl of floral organs, but there were substantial differences in structure and color. The VIGS silencing of *IuAP1* unexpectedly led to the transformation of bracts into leaves, which indicated that the formation of bracts was dominated by *AP1*. The morphogenesis of the posterior sepal is regulated by *AP1*, and the morphological development of the spur was caused by cell proliferation and anisotropic development. Thus, *IuAP1* is involved in the development of the spurs of *I. uliginosa*. *IuAP1* regulates flower color and the formation of speckles to some extent, which is consistent with previous results. This further verifies the functional diversity of *AP1*.

The high levels of expression of *IuAP1* in the posterior sepals may be related to the morphogenesis of posterior sepals and the accumulation of anthocyanins. *IuAP2* was expressed at relatively low levels in the posterior sepal, which could be owing to the inhibition of *IuAP2* expression by some genes that regulate the formation of posterior sepal shape during the process of posterior sepal formation. *IuAP2* was highly expressed in the anterior petals, which could promote the development of the these petals and participated in the first and second rounds of the regulation of floral organ boundaries. The Class A gene and *AG* gene have antagonistic effects (Coito et al., 2018), and they are expressed at very low levels in stamens, even almost not expressed at all, which further confirms this relationship.

#### Class B genes

The levels of expression of the two class B genes was basically the same as those in *A. thaliana*. The *AP3*/*GLO* gene primarily regulates the formation of petals (Theißen and Gramzow, 2016). The silencing or knockout of the *AP3*/*GLO* gene will lead to the transformation of petals to sepals (Hsu et al., 2015). The results of silencing of *IuDEF* showed that it affected the morphogenesis of lateral sepalsand the posterior sepal and anterior petal to some extent. Interestingly, it also regulates the size of the yellow spots in the lateral petals. Yellow pigments are generally related to the accumulation of flavonoid pigments, thus, *IuDEF* may be involved in the biosynthesis of flavonoid pigments. Therefore, to understand how *Impatiens* ensured that its floral organs have different morphogenetic characteristics, it was necessary to conduct a more in-depth study of *Impatiens*. *PI*/*GLO* was considered to be a necessary condition for the development of male flowers. In dioecious plants, the differentiation of male and female flowers depended more on the level of expression of the *PI* gene. Therefore, the *IuGLO* gene could affect the development of stamens and the morphogenesis of *Impatiens*.

#### Class C and D genes

*IuAG* and *IuAGL11* are C and D genes, respectively (Fei et al., 2021). *IuAG* is primarily expressed in the stamens and pistils, which proves that the *AG* gene primarily regulates the development of stamens and pistils. The level of expression of the *IuAG* gene in *I. uliginosa* could be consistent with the function of *AG* gene in most plants (Wang et al., 2015; Su et al., 2018; Liu et al., 2021). *IuAGL11* was primarily expressed in carpels. In our study, we found that the S5 ovules massively proliferated in the carpels. Thus, our results further confirmed that *IuAGL11* was a class D gene that regulates the development of ovules in *Impatiens*.

#### Class E genes

The class E genes had the most complex functions and were involved in the development of almost all the floral organs (Theißen and Gramzow, 2016). Studies have shown that the *SEP* gene plays an important role in the development of floral organs and fruit ripening and can regulate the flavor or ripening period (Li et al., 2017; Qi et al., 2020) of fruits in production. The *SEP* gene also affects the growth and morphology of floral organs (Kaufmann et al., 2009) by regulating the auxin biosynthetic pathway. Therefore, *IuSEP1* and *IuSEP3* were expressed the most highly in the upper wing petal, which indicated that they regulate the morphogenesis of the upper lateral petal. It was hypothesized that *IuSEP1* and *IuSEP3* could inhibit auxin synthesis and caused the morphological development of upper lateral petals to become smaller.

## Conclusions

By comparing the results of our research with those of previous research, the following conclusions were drawn. The *I. uliginosa* flowers took approximately 36 d to develop; the perianth primordia were initiated at approximately 7 d, and the floral organ primordia differentiated at approximately 13 d. The inflorescence of *I. uliginosa* was a spiral raceme. The order of floral organ development was as follows: lateral sepals, posterior sepal, anterior sepals, anterior petal, lateral petals, stamens and gynoecium, and there were four sepals. It was further determined that the cardinal number of floral organs of *Impatiens* was five. The commissure of stamens was caused by the fusion and growth of filament appendages, and the connate filament appendages protected the development of pistil or carpel. The differentiation of each type of organ of *Impatiens* resulted in a state of left and right symmetry of the flower. The formation of bracts in *I. uliginosa* is controlled by *IuAP1*, and *IuAP1* and *IuDEF* are highly diverse functionally, which primarily manifests in the formation of flower color and petal morphogenesis. The combination of analyses of morphological development and gene expression further confirmed that the posterior sepals were part of the first round of floral organs, and the anterior petals were part of the second round of floral organs. The pattern of flower development in *Impatiens* belonged to the ABCDE and tetramer models of flower development, and the function of *I. uliginosa* flower development gene was discussed. This study is highly significant for the study of *Impatiens* flower development and can provide help for the cross-breeding and molecular breeding of *Impatiens*.

## Data availability statement

The datasets presented in this study can be found in online repositories. The names of the repository/repositories and accession number(s) can be found in the article/**Supplementary Material**.

## Author contributions

HHe: Data curation, Formal analysis, Investigation, Methodology, Validation, Visualization, Writing – original draft. XC: Investigation, Validation, Writing – original draft. TW: Investigation, Writing – original draft. ZL: Investigation, Writing – original draft. XZ: Writing – original draft. SQ: Investigation, Writing – original draft. ZG: Investigation, Writing – original draft. MH: Funding acquisition, Supervision, Writing – review & editing. HHu: Funding acquisition, Project administration, Supervision, Writing – review & editing.

## Supplementary material

Supplementary Data Sheet Supplementary Information.

Supplementary Annex 1 gene sequence.

Supplementary Figure 1 I. uliginosa.

Supplementary Figure 2 RNA gel electrophoresis diagram.

Supplementary Figure 3 Flower development model of *Arabidopsis thaliana*.

Supplementary Figure 4 Multiple sequence alignment of ABCDE gene.
