# Supplementary material for: Flower development and a functional analysis of related genes in Impatiens uliginosa
Source: Front Plant Sci. 2024 Mar 25;15:1370949. doi: 10.3389/fpls.2024.1370949 (PMC10999631; doi:10.3389/fpls.2024.1370949)
Supplement: Supplementary Data Sheet — Supplementary Information. [file Table_1.docx]

**>IuAP1**

(5')ATGGGGAGAGGGAGAGTGCAGTTGAAGAGGATAGAGAACAAGATCAATAGGCAAGTTACTTTCTCAAAGAGGAGAGGAGGGCTTCTGAAGAAAGCCCATGAGATCTCCGTACTGTGTGATGCTGAAGTTGCTTTGATCATCTTCTCCCACAAAGGAAAGCTCTTTGAGTACTCTACCGATTCTTGCATGGAAAAGATCCTTGAGCGATACGAGAGGTATTGTTACGCAGAGAGGCGATTGATCAATGATCCAGTTACTATGGGAGACTGGTCCCTGGAATGCAGCAAGCTTAAGGCTAGGATTGAGCTTCTTCAAAGAAACCATAGGCACTACATGGGAGAAGAATTGGATGCCATGAGTATGAGAGAGCTCCAAAACTTGGAGCAGCAACTTGATATTGCACTAAAGCATGTTAGGTCAAGAAAGAATCAACTAATGTTTGAGTCCATTTCTGAGCTGCAAAGAAAGGAGAAAGCCATTTCAGAGCAAAACAGCACCCTAATAAAACAGATCAAGGAGATAGAGAAGACAATGACACAGCAGCAACAGTGGGAGCAGCAAAACCATGATCCAAATGCACCATCCTATCTTATCGGTCAAGACCAACTCCCTTGCCTTAACATTGGAGGAGCTTACCAAGGAGAAGCAGCAATGGAGAGGAGAAACGATCTTGACCTAACGCTTGAACCCGTGGTGTATCCATGTAATCTGAGATGCTTTGCTGCATGA(3')

**>IuAP2**

(5')ATGTGGGATCTCAATGACAACCCCGATCAATCAGACGGCCAAGATTACTCAAAGGGTAAACGAATCGGATCTCTTTCTAACTCCAGCTCGTCGGCATCGGATGCCGCCGCCAGGATATTCGGTTTTCCGATGAACCGACCGGACCCGCCGATGACAAGACAGTTTTTTCCACCGGAGGAGGAGAATATTAATTTTCCTCGGGCGGCGCACTGGGTCGGAGTTAAATTCTACCAATCAGAACTTCCCGTCGGCGGCAAGGGGGTTGAGTCGATTGTTCAGCCTCTAAAGAAGAGTCGCCGGGGACCTAGGTCCCGGAGCTCACAATACAGAGGTGTTACCTTTTACCGGCGAACTGGCCGGTGGGAATCTCATATATGGGATTGTGGGAAACAAGTGTATCTGGGTGGATTTGACACTGCACATGCTGCTGCTAGGGCTTATGATAGAGCTGCCATTAAGTTTCGTGGTGTGGAAGCAGACATAAACTTCACTTTGGAAGAATACCAAGATGACATGAAACAGATGGGTAACTTGACTAAGGAAGAATTTGTGCATGTTCTGAGAAGACAGAGCACTGGTTTTCCAAGAGGAAGCTCCAAATTTAGGGGGGTTACCCTTCACAAATGTGGCAGATGGGAAGCCAGAATGGGTCAATTCTTGGGCAAAAAGTAAATCTATTTTTCACTTTCTTTTTCTATCTCTCTGTATTGA(3')

**>IuDEF**

(5')ATGGCCAGAGGAAAGATCCAGATCAAGAGAATCGACAACGATACTAACAGGCAGGTAACCTACTCTAAGCGCCGTAACGGACTCTTCAAGAAAGCCGGCGAACTTACTGTGCTATGCGATGCTAAGGTCTCCATCATCATGCTCTCAAGCACCGGCAAACTCCATGAATACATCAGCTCCTCCATTTCGACGAAACAGCTGTTTGATCACTACCAGAACACACTGGCGGTTGATCTTTGGAGTTCAAAGTATCAGAGAATGCAAGAGCATTTGAAGAAACTAAAAGAAGTGAATAGACTTCTTCGAATGGAGATTAGCCAGAGGATGGGTGAGAATCTGAGTCACCTTTGCTATGAAGAAGTGACGAAACTCGAACAAGATGTGGACAGTTCCTTGCAGCAAATCCGAGACCGTAAGTTTAAGGTGCTTGGAAACCAGATTGAGATTCACAAGAAGAAGGTGAGTTGA (3')

**>IuGLO**

(5')ATGGGGAGAGGAAAGATTGAAATAAAGAGGATAGAGAATTCAAGCAACAGACAAGTCACATATTCAAAGAGGAGAAATGGTCTAATCAAGAAAGCTAAAGAAATCACCGTTCTTTGTGATGCTAAGGTTTCTCTTGTCATCATCGGAAGCTCCGGCAAGATTCACGAGTATTGCAGCCCATCTACAACTTTACCAGATATCCTTGAAAGATATCACAAGCAGTCTGGAAAGAAGCTATGGGATGCCAAGCATGAGAACCTGAGCAATGAGATTGATAGAATCAAGAAAGAGAATGACAATATGCAGATTGAGCTAAGGCATCTAAAAGGAGAAGACATTACATCTTTGCACTACAAAGAGCTCATGGCCTTGGAAGATGCACTTGAAAATGGCCTAATTGGAGCTCGCGAAAAACAGATGGAGATCTACAAGATGATGAAGAAAAATGATAGGATTTTGGAGGAGGAGCACAAGGAGCTAAATTTTGTGCTGCAACAAAGAGAGATGGCTATGGAACGTAGAGAAATGGAACATAACGGCTACCACCAGCAAATGCAAGATTTTGAGCCTCAGATGCCTTTCACCTTCCGAATCCAGCCAATGCATCCAAATCTGCAAGAGAGAATGTAA(3')

**>IuAG**

(5')ATGGCTTTGCCAAGTGAATCTATGGCGCTTCTTGATTTATCTGGACAGAGAAAGAACGGGAGAGGAAAGATAGAGATAAAGAGGATTGAAAACACCACCAATCGACAAGTTACCTTCTGCAAACGTAGGAATGGATTGTTGAAGAAAGCCTATGAATTATCTGTTCTTTGTGATGCTGAGGTGGCACTTGTTGTCTTCTCCAGCCGCGGTCGTCTCTACGAGTATGCTAATAACAGTGTTAGGGGCACAATTGAGAGGTACAAAAAAGCATCCTCTGATTCTCCAAATACTGCTGGCTCTGTAGCTGAAGCCAATGCTCAGTTTTACCAACAAGAATCCTCCAAATTGCGACAACAAATCGGCAATTTGCAGAATTCAAACAGGCAAATTCTCGGCGAATCTTTGGGTTCTATGAGTCTTAGGGATCTTAAGAGTCTTGAAAGTCGTTTAGAGAGAAGCATTAGCAAGATTCGTTCTAAAAAGAATGAGTTGTTGTTCGCTGAGATCGACTTCATGCAAAAGCGGGAAGTTGACTTACATAACAATAATCAATTCCTAAGAGCTAAGATTTCCGAGAGTGAGAGAGCCCAACAACAACAACAACCTCAGATGAGTTTAATGCCAGGAGGATCTAACTACGACCAGCTGGTTCAACCTCAGCCGTCATCTTTCGACAATAGAAACTTCTTTCAGGTTACCGCGTTGCAACCTGATAATCAGTATTCTCGTGATGATCAGACTCCGCTTCAGCTTGTTTAA (3')

**>IuAGL11**

(5')ATGGGTCGTGGAAAGATAGAGATAAAGAGGATTGAAAACAACACAAACAGACAAGTAACGTTCTGCAAACGTAGGAATGGACTACTAAAGAAGGCTTATGAATTGTCTGTTTTGTGTGAAGCTGAGGTTGCACTTATTGTCTTCTCTAGTCGTGGCCGTCTCTACGAGTATGCCAATAACAACATAAGATCGACTATAGAGAGGTACAAGAAGTCTTGCTCAGAAACAACTAATCCAATCCCCACTCCTGAGATCAATGCCCAATTCTATCAACAAGAGTCAAAGAAGCTTCGCCAACAAATTCAAGGGCTTCAGAACACCAACAGGAATCTAGGTGGAGAAGGATTGGGATGCTTGAACTTAAAGGATTTGAAACAGCTTGAAAATAGGCTTGAGAAAGGCATCTCAAGAATCAGATCCAAAAAGCATGAGCTAATACTAGCAGAAACTGAATGCTTGCAGAAAAGGGAGATAGAGCTGGAACATGAAAATGCATGCCTTAGAGCCAAGATTGCCGAAAGTGAGAGGATTCAGCAGCAGCTAGGCATGTTGCAGGGACAAGAATACATGATGAACAATGCTGCGATGCATTTGCACGCATATTTCTCTCGCAATGTTCTTCTCATGAACAACAATATCATTAATAATAACTTAAACAATGATCAAGAGGAGGCTGCTGGTCAAGATGATGAAGGTGGTGCTTCCCTACCATATCGACCCCTCCTAATCCCCGACAACAAGTCTCTTCTTCATCTTGGATAG(3')

**>IuSEP1**

(5')ATGGGAAGGGGAAGATTGGAGCTGAAGAGGATAGAGAACAAAATAAATAGGCAAGTTACGTTTGCAAAAAGGAGGAATGGGGTGCTCAAGAAAGCCTATGAGCTCTCTGTTCTTTGTGATGCCGAGGTTGCTCTCATCATCTTCTCCAACAGAGGGAGGCTCTATGAGTTCTCCAGTTCATCCAACATGCTCAAAACTCTTGAAAGATACGAAAAATGCAGTTATGTGACACCTGAAGTTAACCAAGCAGCCAAAGAAATTGAGCAAAGTAGCTATAGAGAATACCTGAAACTGAAAGCAAAATATGATGCACTACAACTTCATCAGAGGAATCTTCTTGGCGAAGACCTGGGCCCACTGAATGTTAAGGATCTTGAACATATTGAGCTTCAACTTGATTCATCTCTCAGGCATGTTCGATCAACCAAGACCCAACTGATGCTTGATCAGTTTTCTGATCTTCAAACAAAGGAGAAAGCATTGGTTGAAGCTAACAGGGCTTTGGAAAGAAAACTAGACGAAATGTACGGACAGATGCAACTTCAGCCGCCACCATGGCCAGGCAGCAGCGATCAAGGCAGTAGTTCATTATATGCTCAGCCAGTACACCATCCTCCTCCTCAATCTCAAGGGTTTTTCCAGCATCTTGATTGCAACTTCAATTTACAAATCGGGTATGATCCAACAGGAGGAGGAGGAAGCAGGATAGCTGCTGCTGCAACTCAAGAACAAAATGTGAATGGTTTGATGCCAGGTTGGATGCTCTGA(3')

**>IuSPE3**

(5')ATGGGCAGGGGTAGGGTTGAGCTAAAGAGGATAGAAAACAAGATCAACAGACAGGTAACCTTTGCGAAACGAAGAAACGGTCTGTTGAAGAAAGCTTACGAACTTTCTGTTCTCTGCGATGCCGAGGTAGCTCTCATCATCTTCTCCAACCGAGGAAAACTCTATGAGTTTTGCAGTAGTCCCAGCATGCTGAAAACACTCGAGAGATACCAAAAATGTAACTACGGCGCACCTGAACCAAATGTTTCGGCAAGGGGATCTTTGGAACTAAGTAGTCAACAAGAATATTTGAAACTCAAACAACGCTATGAAGCCTTACAAAGAACCCAAAGGAATCTTTTGGGAGAGGATCTCGGTCCTTTAGACGGCAAGGAACTCGATGCAATTGAGAGACAACTCGACATGTCATTGAAGCATATCAGATCAACACGTACGCAATATATGGTTGATCAACTCGCGGACTTGCAAAGAAAGGAGCATCTTCTCAACGAAGCAAACAGGGCCTTGAAACAAAGGCTGTTGGAAAGTTCATTCAATTGGATGCAAAATGGGCCAGATTTGGATTATAGTGGACAACCTGTTCAGCCCAATGGAGATGCTTTCTTTCATCCATTGGACTGTGAGCCCACCTTACAAATGGCAATGGGGTATCAAACACATGATCATGATCCATCAACAGTGGAAGTTGCTGGTCCAAGTATGAACAACTACTTTCCAGGATGGCTGCCATAA(3')
