# Supplementary material for: Flower development and a functional analysis of related genes in Impatiens uliginosa
Source: Front Plant Sci. 2024 Mar 25;15:1370949. doi: 10.3389/fpls.2024.1370949 (PMC10999631; doi:10.3389/fpls.2024.1370949)
Supplement: Supplementary Annex 1 — gene sequence. [file Table_2.docx]

**1 Statistics of the length of flower development**

**Table 1 Flower development stage**

| **Period** | **Interval time**(d) | **Period time**(d) |
| --- | --- | --- |
| S1 | / | 0 |
| S2 | 6.78±0.83 | 7 |
| S3 | 5.78±0.83 | 13 |
| S4 | 6.33±1.12 | 19 |
| S5 | 7.22±0.44 | 26 |
| S6 | 4.89±0.78 | 31 |
| S7 | 3.33±0.5 | 34 |
| S8 | 1.56±0.52 | 36 |

**2 Fluorescent quantitative primers**

**Table 2 Primers sequence table**

| N | Name | Product length | Primer sequence | Length | Tm | GC% |
| --- | --- | --- | --- | --- | --- | --- |
| 1 | IuAP1 | 141 | F: ACCATAGGCACTACATGGGA | 20 | 57.81 | 50 |
|  |  |  | R: GCAGCTCAGAAATGGACTCA | 20 | 57.89 | 50 |
| 2 | IuAP2 | 190 | F: CGCCAGGATATTCGGTTTTC | 20 | 56.96 | 50 |
|  |  |  | R: GCGACTCTTCTTTAGAGGCT | 20 | 57.05 | 50 |
| 3 | IuDEF | 133 | F: TATGCGATGCTAAGGTCTCC | 20 | 56.89 | 50 |
|  |  |  | R: GAACTCCAAAGATCAACCGC | 20 | 57.1 | 50 |
| 4 | IuGLO | 109 | F: GATTCACGAGTATTGCAGCC | 20 | 56.97 | 50 |
|  |  |  | R: ATTGCTCAGGTTCTCATGCT | 20 | 57.2 | 45 |
| 5 | IuAG | 140 | F: GAGGTGGCACTTGTTGTCTT | 20 | 58.32 | 50 |
|  |  |  | R: GCATTGGCTTCAGCTACAGA | 20 | 58.26 | 50 |
| 6 | IuAGL11 | 191 | F: GAAGCTGAGGTTGCACTTAT | 20 | 55.74 | 45 |
|  |  |  | R: CCTTGAATTTGTTGGCGAAG | 20 | 55.84 | 45 |
| 7 | IuSEP1 | 164 | F: TATGCTCAGCCAGTACACCA | 20 | 58.43 | 50 |
|  |  |  | R: CCTGGCATCAAACCATTCAC | 20 | 57.34 | 50 |
| 8 | IuSEP3 | 173 | F: TCTCCAACCGAGGAAAACTC | 20 | 57.17 | 50 |
|  |  |  | R: GGCTTCATAGCGTTGTTTGA | 20 | 56.73 | 45 |
| 9 | Reference gene | / | F: TGAATGTCCCTGCTGTTTG | 19 | 55.71 | 47.37 |
|  |  |  | R: ACCTTCCGCATAACTTTACC | 20 | 55.44 | 45 |

**3 Expression analysis**

**Table 3** relative expression of floral development genes

| G  P | IuAP1 | IuAP2 | IuAP3 | IuGLO | IuAG | IuAGL11 | IuSEP1 | IuSEP3 |
| --- | --- | --- | --- | --- | --- | --- | --- | --- |
| Ls | 54.32±1.08 | 106.35±4.08 | 1.01±0.04 | 0.0025±0.00 | 0.08±0.04 | 0.10±0.00 | 0.41±0.08 | 6.81±0.53 |
| Ps | 112.52±4.4 | 6.38±0.27 | 3.00±0.12 | 1±0.00 | 0.26±0.03 | 1.78±0.20 | 9.70±1.13 | 51.50±2.82 |
| Ap | 35.42±0.51 | 41.32±0.87 | 26.97±3.23 | 34.54±0.41 | 0.08±0.03 | 1.09±0.07 | 9.96±0.21 | 22.13±1.72 |
| Ulp | 50.71±2.16 | 8.65±0.21 | 37.97±3.20 | 76.90±4.54 | 1.10±0.09 | 0.34±0.05 | 115.38±6.78 | 117.55±7.62 |
| Llp | 67.17±0.93 | 22.04±0.66 | 71.39±6.73 | 105.74±5.04 | 0.18±0.01 | 0.14±0.03 | 18.10±3.02 | 55.28±12.4 |
| St | 0.92±0.07 | 4.97±0.29 | 22.32±0.15 | 34.04±2.36 | 62.94±4.58 | 0.79±0.01 | 5.70±0.65 | 0.35±0.03 |
| Ca | 0.33±0.02 | 11.43±1.22 | 13.67±1.83 | 7.97±0.77 | 108.80±7.29 | 123.30±14.0 | 17.07±0.88 | 36.84±4.67 |
