# Supplementary figures and images for: Flower development and a functional analysis of related genes in Impatiens uliginosa
Source: Front Plant Sci. 2024 Mar 25;15:1370949. doi: 10.3389/fpls.2024.1370949 (PMC10999631; doi:10.3389/fpls.2024.1370949)

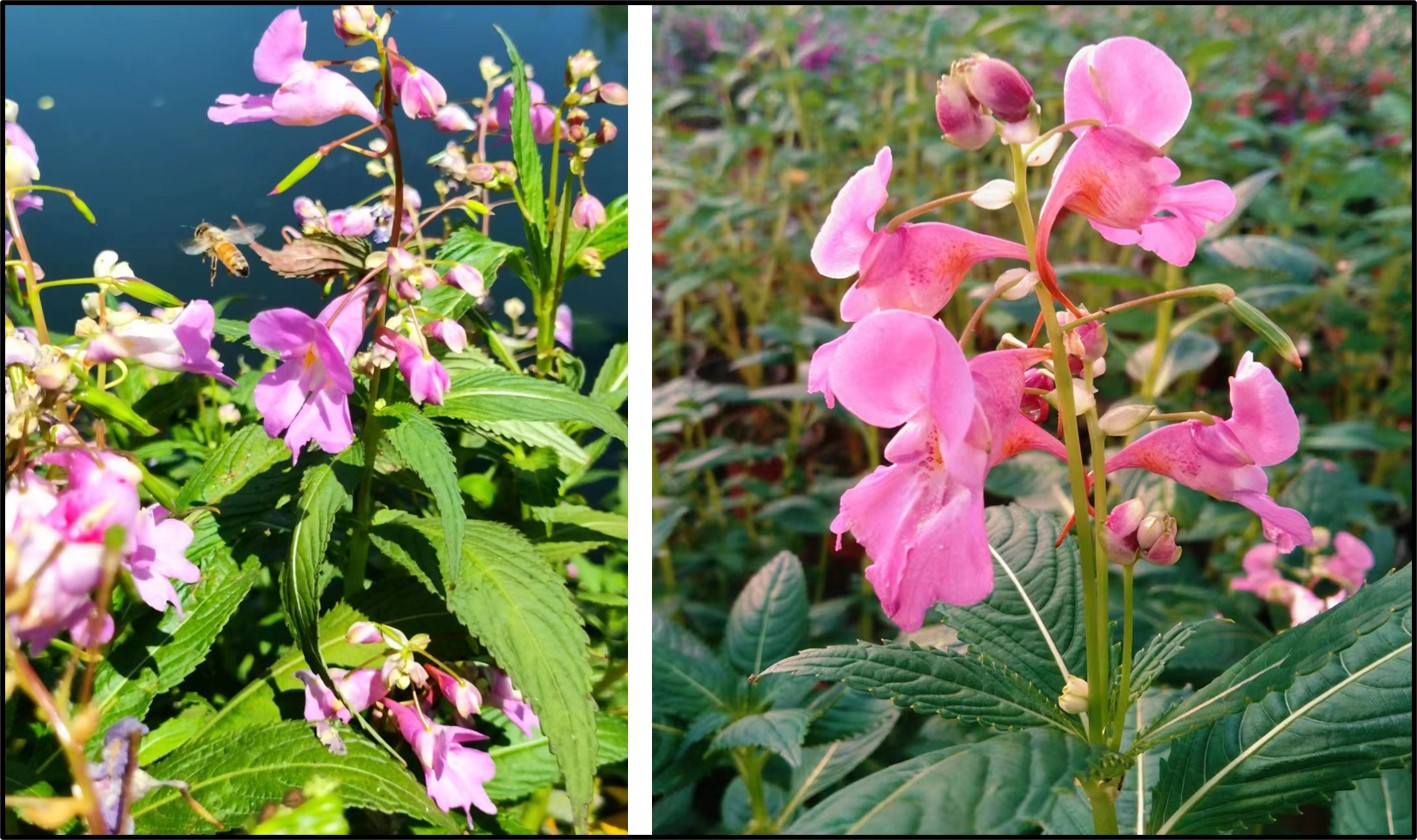

Supplement: Supplementary Figure 1 — I. uliginosa. [file Image_1.jpeg]

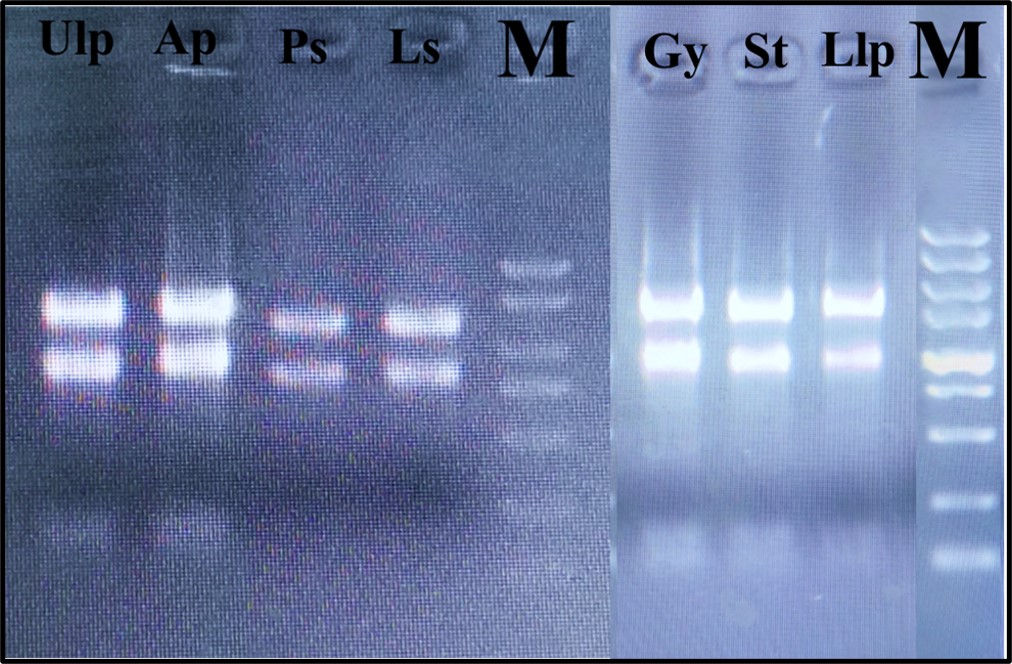

Supplement: Supplementary Figure 2 — RNA gel electrophoresis diagram. [file Image_2.jpeg]

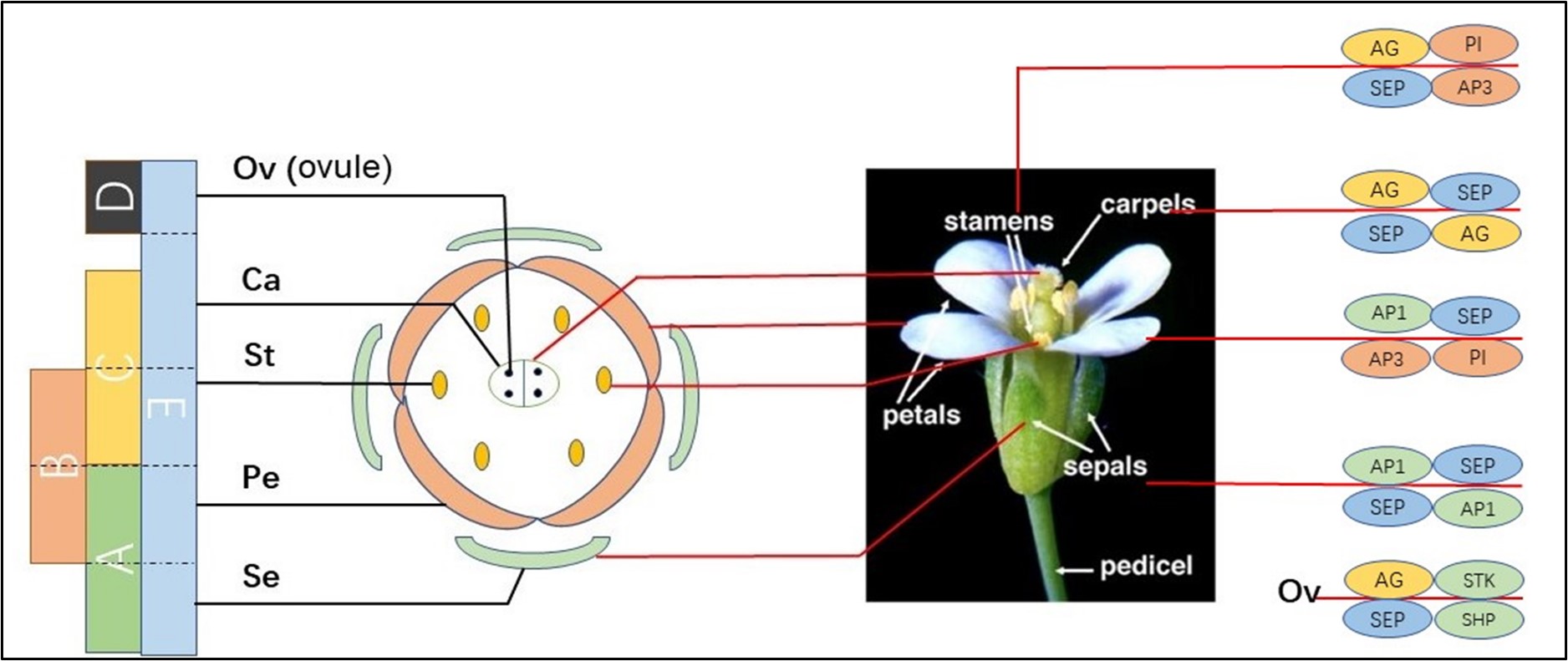

Supplement: Supplementary Figure 3 — Flower development model of Arabidopsis thaliana. [file Image_3.jpeg]

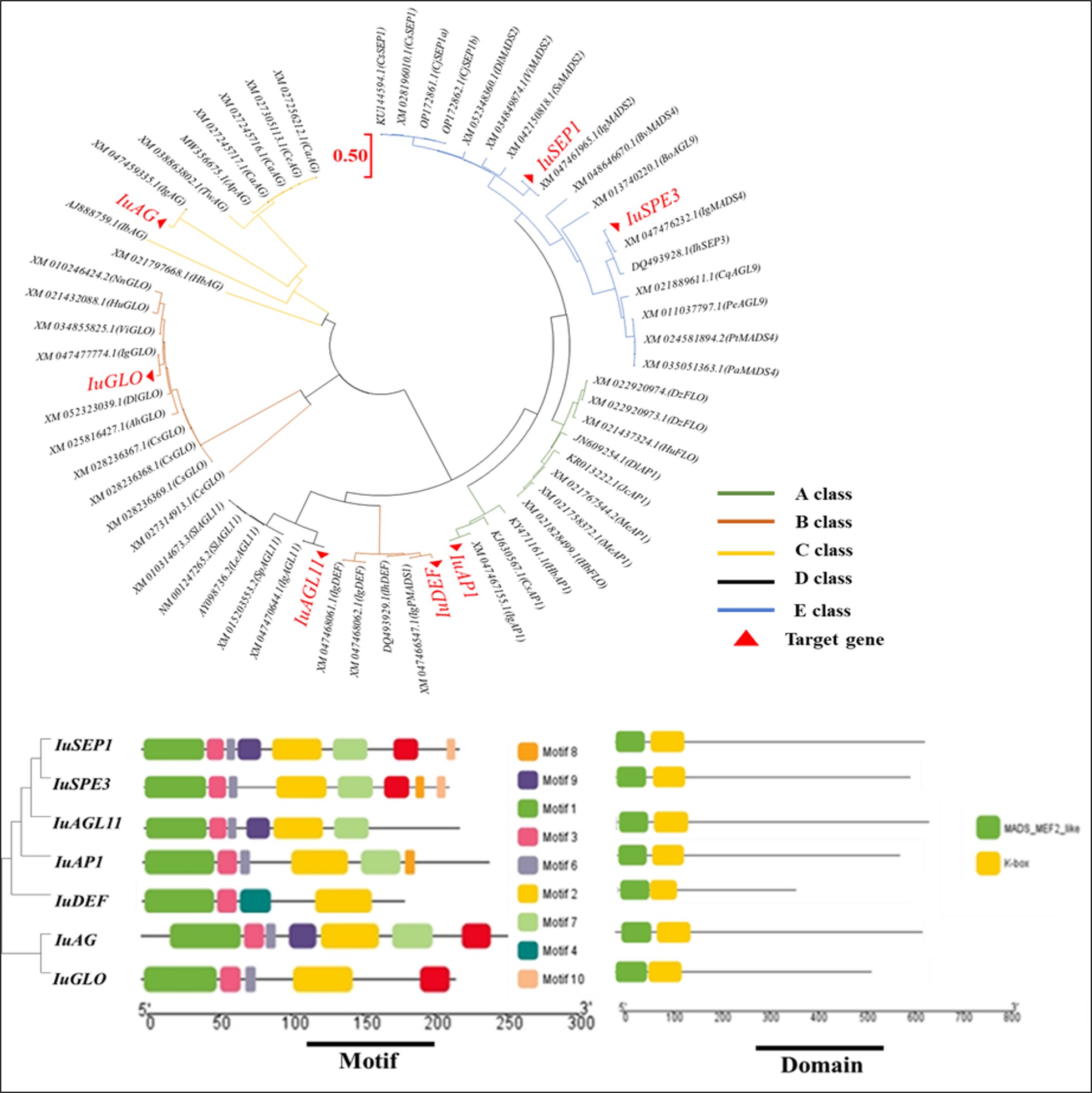

Supplement: Supplementary Figure 4 — Multiple sequence alignment of ABCDE gene. [file Image_4.jpeg]
